# Supplementary material for: The interactome of intact mitochondria by cross-linking mass spectrometry provides evidence for coexisting respiratory supercomplexes
Source: Mol Cell Proteomics. 2017 Dec 8;17(2):216–32. doi: 10.1074/mcp.RA117.000470 (PMC5795388; doi:10.1074/mcp.RA117.000470)
Supplement: Supplemental Data [file supp_RA117.000470_133922_0_supp_23978_fzffwf.zip › spectra_annotation/mito_DR_spectra_annotation/41-1-8-1-4-1.pdf]

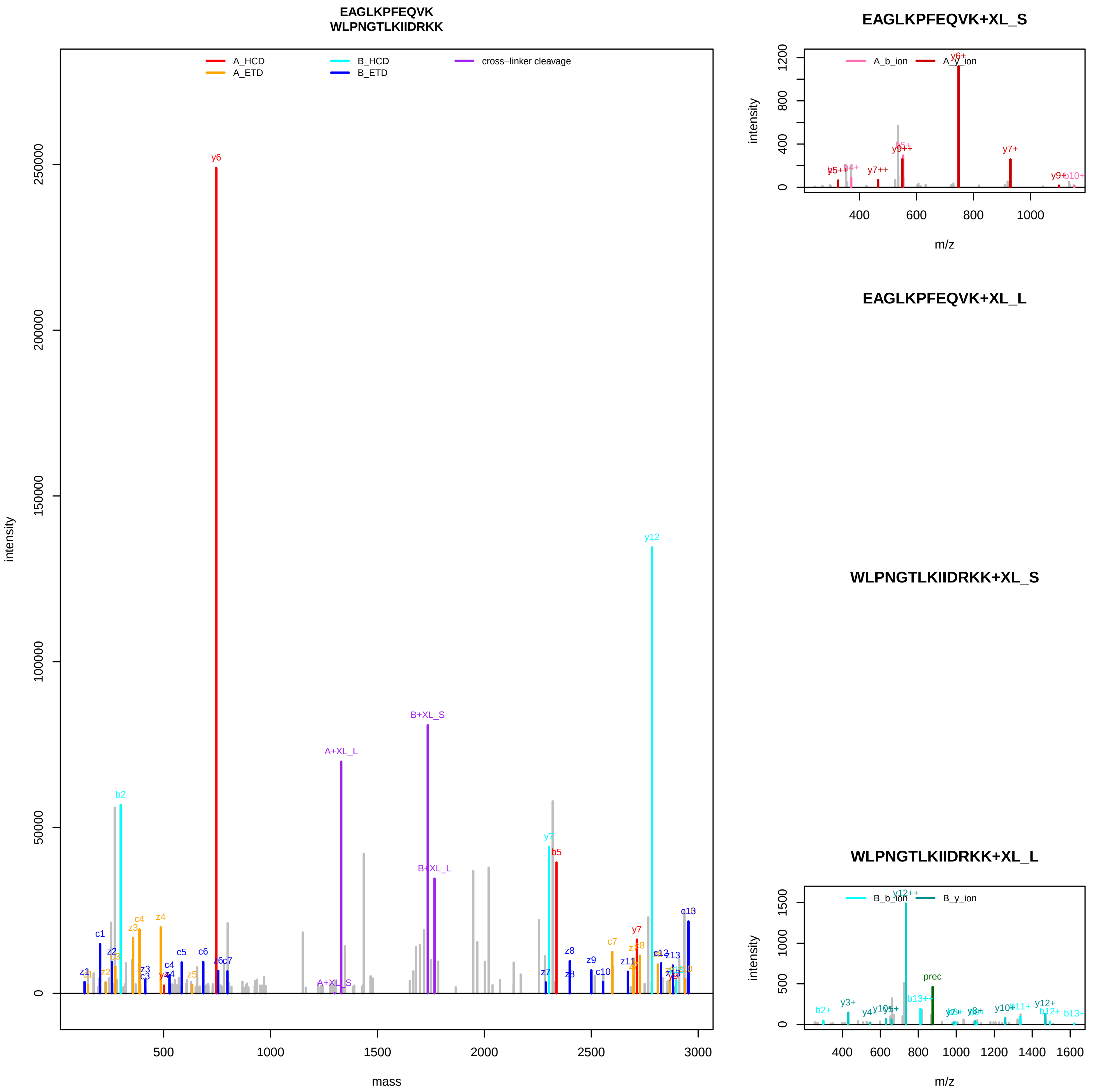

EAGLKPFEQVK
WLPNGTLKIIDRKK
A_HCD
A_ETD
B_HCD
B_ETD
cross−linker cleavage
intensity
mass
0
50000
100000
150000
200000
250000
500
1000
1500
2000
2500
3000
y6
y12
B+XL_S
A+XL_L
B+XL_L
A+XL_S
b2
y7
b5
c13
EAGLKPFEQVK+XL_S
A_b_ion
A_y_ion
y6+
y7+
y9++
y7++
y5++
y9+
b10+
intensity
m/z
0
400
800
1200
400
600
800
1000
EAGLKPFEQVK+XL_L
WLPNGTLKIIDRKK+XL_S
WLPNGTLKIIDRKK+XL_L
B_b_ion
B_y_ion
y12++
prec
b13++
b2+
y3+
y4+
y8+
y10+
b11+
y12+
b12+
b13+
intensity
m/z
0
500
1000
1500
400
600
800
1000
1200
1400
1600
